# Can the predictive value of multiparametric MRI for prostate cancer be improved by a liquid biopsy with SelectMDx?

**DOI:** 10.1002/cnr2.1396

**Published:** 2021-05-01

**Authors:** Mohammad Sajjad Rahnama'i, Christian Bach, Maximilian Schulze‐Hagen, Christiane K Kuhl, Thomas Alexander Vögeli

**Affiliations:** ^1^ Department of Urology Uniklinik RWTH Aachen Germany; ^2^ Department of Radiology Uniklinik RWTH Aachen Germany

**Keywords:** biomarkers, mpMRI, prostate cancer, SelectMdx, urinary marker

## Abstract

**Background:**

SelectMDx is a urinary biomarker test for determining prostate cancer risk.

**Aim:**

In a group of patients with a biopsy proven prostate cancer (PCa) who had undergone a multi parametric Magnetic Resonance Imaging (mpMRI) and urinary biomarker test with SelectMDx, we studied the additive value of SelectMDx to mpMRI and correlated that to the radical prostatectomy histology.

**Methods and results:**

Thirty‐nine consecutive patients with a positive prostate biopsy were included in the study. They all had mpMRI and SelectMDx and underwent a radical prostatectomy. Overall, the mpMRI showed a PIRADS ≤3 lesion in seven cases out of the 39 patients. Significant lesions (PIRADS ≥4) were found in 32 cases (82%), that is, in 17 cases a PIRADS 5 lesion and in 15 cases a PIRADS 4 lesion. The mpMRI missed significant PCa in seven cases (18%) who had a PIRADS ≤3 lesion but had a significant PCa on final histology after RP. In our study, the positive predictive values of mpMRI were 97% and that of the SelectMDx was 100%.

**Conclusion:**

In this real‐life selected group of consecutive patients with a confirmed positive PCa biopsy and available mpMRI, the liquid biopsy test with SelectMDx, did not provide an additional information about the PCa clinical significance. The addition of SelectMDx was only found valuable in those patients who had a very high‐risk PCa (ie, GS ≥8) who had a positive SelectMDx test outcome despite of a negative mpMRI outcome.

## 1 INTRODUCTION

Although most prostate cancer (PCa) is currently diagnosed through prostate‐specific antigen (PSA) screening, this test is not specific for clinically significant PCa. Moreover, false positives trigger unnecessary biopsies, and biopsy risks have increased. In addition, PCa represents a wide spectrum of disease, ranging from clinically indolent to aggressive, high‐grade cancers. PSA‐based screening leads to overdiagnosis and overtreatment.^1^ Hence better markers are needed to diagnose clinically significant PCa. There are several PCa marker tests available that are derived from blood, urine, and tissue.^1^

Multiparametric magnetic resonance imaging (mpMRI) of the prostate has been introduced to improve the detection of clinically significant PCa. There is an ongoing discussion on the utility of mpMRI screening before transrectal ultrasound‐guided biopsy.^2^

In addition, a three‐gene panel urine test using HOXC6, TDRD1, and DLX1 was recently introduced to assist in the diagnosis of Gleason ≥7 PCa. This urine marker test is made commercially available in some European countries and the United States under the name of SelectMDx.^3^

SelectMDx is a biomarker test that measures urinary RNA levels of two genes (DLX1 and HOXC6) following digital rectal examination (DRE). Using an algorithm including tPSA, PSA density, DRE, age, and family history, it provides an individual patient's likelihood of low‐ and high‐grade PCa. This tool was developed after a study in 519 men undergoing prostate biopsy and was subsequently validated in a cohort of 386 men^4^. SelectMDx demonstrated an AUC of 0.86 (95% CI 0.80‐0.92) for high‐grade PCa and outperformed the base model without RNA markers and the Prostate Cancer Prevention Trial Risk Calculator (PCPTRC).

SelectMDx liquid biopsy has been added to the most recent 2018 update of the guidelines of the European Association of Urology (EAU) as a possible tool in the diagnostic workup of the men being considered for prostate biopsy.^4^

In this study, we present real‐life data aiming to determine if the predictive value of mpMRI can be improved by a liquid biopsy.

## 2 MATERIAL AND METHODS

Between July 2018 and November 2020, we prospectively identified all patients who had undergone a mpMRI before their transrectal prostate biopsies and had additionally undergone a liquid biopsy test (SelectMDx). From this group of patients, those who had undergone a radical prostatectomy were included in our study.

The criteria for the transrectal prostate biopsy included a repeated measurement of an elevated PSA level > 4 ng/mL and/or an abnormal DRE. All biopsies were carried out by either of the two urologists of our department.

In all included patients with ≥ PIRADS 3 lesion, transrectal MRI‐Ultrasound fusion biopsy was carried out under local anesthesia in addition to a regular sextant biopsy. All patients how had a ≤PIRADS 3 lesion on their mpMRI, underwent a regular transrectal sextant biopsy. All patients with a transrectal biopsy outcome that was negative for PCa as well as patient who did not undergo a radical prostatectomy, were excluded from our study.

Urine was collected of patients in our outpatient clinic during their preoperative diagnostic work up for PCa. Approximately 30 mL of first voided urine was collected in a collection cup after DRE. Urine was immediately transferred into a urine specimen transport tube (Hologic Inc), and samples were shipped at room temperature to a central laboratory and stored within at −80°C.

The tests were carried out by SelectMDx laboratory in the Netherlands and carried out as described by Van Neste et al. In short, fixed whole urine was used as substrate to further optimize and standardize the assay. Assays were performed using a prototype amplification kit (Labo Biomedical Products BV, Rijswijk, The Netherlands). In short, RNA was isolated out of 1 mL urine using the MagNA Pure 96 instrument (Roche Life Science, Indianapolis, IN, USA). Subsequently, RNA levels of HOXC4, HOXC6, TDRD1, DLX1, KLK3, and PCA3 were determined using one‐step reverse transcription quantitative polymerase chain reaction. The KLK3 gene, encoding for PSA, is a kallikrein serine protease and used as a reference for relative biomarker quantitation using the DDCt method.^5^

All of the included patients had undergone a robotic radical prostatectomy (RP) with bilateral pelvic lymph node dissection using the Da Vinci Xi system and were operated by the same surgeon.

The arithmetic percentages of the positive negative test outcomes were calculated. In addition, the positive predictive value (PPV) of the diagnostic tests could be calculated.

## 3 RESULTS

Between July 2018 and November 2020, all patients who had a prostate cancer diagnosis confirmed by transrectal biopsy and had a liquid biopsy test by SelectMDx as well as mpMRI were identified. Only those patients who subsequently underwent an RP were included in our study. Thirty nine consecutive patients were identified (mean age 66 years range: 45‐77 years) who fulfilled our inclusion criteria and were included in our study.

Twenty‐one patients had an abnormal DRE. The initial PSA ranged between 0.5 and 66.78 ng/mL. The outcome of the SelectMDx test as well as the mpMRI and the histology report of the radical prostatectomy specimen are listed in Table 1. The RP histology of most of the included patients showed a GS of 3 + 4 (n = 20).

**TABLE 1:** The Gleason score (GS) and the outcomes of SelectMDx test and mpMPR of the included patients

No Patients N = 39	Gleason Score in the RP Histology	SelectMDx Risk for GS ≥7 outcome: very low or < 50%	SelectMDx Risk for GS ≥7 outcome: ≥ 50%	mpMRI PIRADS ≤3	mpMRI PIRADS 4	mpMRI PIRADS 5
1	3 + 3	1	‐	‐	1	‐
20	3 + 4	16 (80%)	4 (20%)	4 (20%)	10 (50%)	6 (30%)
11	4 + 3	6 (54%)	5 (45%)	0	3	8
2	4 + 4	1	1	1	1	‐
3	4 + 5	‐	3	1	‐	2
1	5 + 4	‐	1	1	‐	‐
1	5 + 5	1	‐	‐	‐	1

The characteristics of the mpMRI and SelectMDx test in our study population are summarized in Table 2.

**TABLE 2:** The positive predictive value (PPV) of the mpMRI and SelectMDx test

	PPV
mpMRI	97%
SelectMDx	100%
mpMRI + SelectMDx^a^	100%

^**a**^ In the calculations for the combination of mpMRI and SelectMDx test, a negative SelectMDx result and a positive mpMRI result was considered a negative test outcome.

Overall, the mpMRI showed a negative outcome (PIRADS ≤3 lesion) in seven cases. Significant lesions were defined as PIRADS ≥4 and were found in 32 cases (82%) (in 17 cases PIRADS 5 and in 15 cases PIRADS 4).

A total of 31 patients had a PRIADS 4 or 5 lesion and a Gleason 3 + 4 or higher in their RP histology report. Therefore, true positive mpMRI test outcomes were 31 (value A).

Only one patient had a PIRADS 4 lesion and a Gleason 3 + 3 in the RP histology. Hence, the false positive result for mpMRI was 1 (Value B). The positive predictive value (PPV) of the mpMRI in our study, calculated as value A/(value A + value B) × 100 is 97%.

None of the included patients in our study had a mpMRI outcome of PIRADS ≤3 with a none significant PCa or benign histology in the final histology. This makes that, the true negative mpMRI test outcome was 0. On the other hand, the mpMRI missed significant PCa in seven cases (18%) who had a clinically significant PCa in the RP histology report despite a PIRADS ≤3 outcome in the mpMRI. Therefore, the false negative mpMRI test outcome in our series was 7.

SelectMDx test data showed that from the included 39 patients, 25 patients (64%) had a liquid biopsy outcome for GS ≥7 PCa of either very low (stated as very low‐risk and no percentage mentioned in the test outcome) or a low‐risk defined as a risk percentage of <50% for a significant Pca (ie, GS ≥7) diagnosis on biopsy. This means that in those 25 patients the SelectMDx outcome was considered negative.

A total of 14 patients had a positive SelectMDx test outcome defined as a ≥ 50% chance of GS ≥7 PXa risk. All of these 14 patients had a RP histology of GS ≥7. Hence the true positive value of the SelectMDx test in our series was 14 (value A). There were no false positive SelectMDx test outcomes (value B). The positive predictive value (PPV) of the SelectMDx test in our study, calculated as value A/(value A + value B) × 100 is 100%.

On the other hand, as mentioned above, 25 patients had a negative SelectMDx test outcome. One of these patients had indeed a GS 3 + 3 in the RP histology report. Hence, the true negative value of the SelectMDx test is 1 and the false negative value is 24.

## 4 DISCUSSION

New promising PCa‐specific biomarkers have been identified in many studies.^3^, ^4^ Nevertheless, to date, only a few biomarkers have reached clinical practice. The challenge remains to validate the real‐life utility and performance of the biomarkers in a clinical practice.

In the last few years, mpMRI has shown growing relevance in pre‐biopsy diagnosis of PCa.^6^ In this context, it could be interesting to evaluate how biomarkers such as SelectMDx could be combined with mpMRI. To date, there have been no studies evaluating the additive value of SelectMDx to mpMRI with radical prostatectomy histology confirmation.

### 4.1 SelectMDx test

In our real‐life study of 39 patients, we found that, 25 patients (64%) had a liquid biopsy SelectMDx test outcome for GS ≥7 PCa of either very low (stated as very low‐risk and no percentage mentioned in the test outcome) or a risk percentage of <50%. From these 25 patients, one patient had a GS 3 + 3 and 16 patients had a GS 3 + 4. The other 8 patients had a GS 4 + 3 (6 pts) GS 4 + 4 (1 pt) and even 5 + 5 (1 pt), respectively. So it can be concluded that SelectMDx test is not a sensitive test for GS 3 + 4 and can also miss patients with high‐risk PCa.When considering the overall value of the SelectMDx test we can state that with a cut off value of GS ≥7 as significant PCa the test has a false negative rate of 62% (24 out of 39 cases) and hence is not a valuable tool. With a cut off GS ≥8 the false negative rate is much less that is, 5% (2 out of 39 cases).The positive predictive value (PPV) is the probability that subjects with a positive screening test truly have the disease. In our study, the SelectMDX test had a PPV of 100% (Table 2).

### 4.2 mpMRI

By using mpMRI, clinicians could significantly reduce the number of unnecessary repeat prostate biopsies.^7^ Unnecessary biopsies can be reduced by about 50% when a PIRADS score of 3 or greater is used as a cut off, taking in mind a rate of 16.2% and 39.7% false‐negative rates of clinically significant PCa for targeted fusion prostate biopsy of PIRADS 3 or greater and PRADS 4 or greater lesions, respectively.^7^

In the 39 included patients of our study, the mpMRI missed significant PCa (ie, GS ≥7 PCa) in 18% of the cases. In these seven cases, PCa was found despite a PIRADS ≤3 mpMRI outcome. Three of these patients had a GS 8 or 9 in their RP histology and four patients had GS 3 + 4 after RP. (Table 1) The PPV of mpMRI was 97%.

### 4.3 Combination of SelectMDx and mpMRI

A plausible scenario would be that subjects with PIRADS 5 lesions should proceed to biopsy directly, whereas for PIRADS 4 lesions, SelectMDx should be taken to select patients who really would need a biopsy. This could be particularly relevant since data in the literature have demonstrated a wide range of negative predictive values for clinically significant PCa identification (63%‐98%),^8^ leading to a high rate of repeated unnecessary biopsies in men with negative mpMRI. In such a context, a non‐invasive test such as SelectMDx could be very useful to choose the patients who will benefit most from biopsy, avoiding the risk for patients and the waste of money.

In a retrospective study that evaluated the correlation of SelectMDx test with mpMRI, the median SelectMDx score was significantly higher in patients with a suspicious significant lesion on mpMRI compared to no suspicion of significant PCa and there was a positive association between SelectMDx score and the final PIRADS grade.^9^ In addition, the authors found a statistically significant difference in SelectMDx score between PIRADS 3 and 4 and between PIRADS 4 and 5.^9^

In a more recent study, the SelectMDX liquid biopsy results of test 45 patients with low‐risk PCa who were under active surveillance with were evaluated.^10^ A total of 9/45 (20%) of these patients were reclassified from low‐risk to clinically significant PCa.^10^ In this selective cohort of low‐risk patients under active surveillance the authors found a PPV for mpMRI of 54% vs 28% for SelectMDx as well as an NPV of 92% for mpMRI vs 87% for SelectMDX.^10^

In our study, when adding the liquid biopsy SelectMDx risk outcome in the seven patients with negative mpMRI, it turns out that the SelectMDx test had reported a low‐risk for significant PCa in four of these cases. These four patients had a GS 3 + 4 in their report. The SelectMDx test was valuable in the remaining three cases with a negative mpMRI, where it showed an increase risk for clinically significant PCa (ie, a risk of 92% in a patient with GS 5 + 4, 52% in a patient with GS 8 and 91% in a patient with GS 4 + 5).

Therefore, the addition of SelectMDx was only valuable in those patients with very high‐risk PCa (ie, GS 9) and a negative mpMRI. Hence, for patients with GS 3 + 4, a combination of mpMRI and SelectMDx test did not offer any advantage.

When combining SelectMDx and mpMRI outcome in a way that a negative mpMRI and a positive SelectMDx test result would be considered a positive test outcome and lead to a transrectal biopsy and a negative SelectMDx and a positive mpMRI would be considered a negative outcome, the PPV of the combination of mpMRI and SelectMDx test will be 100%.

A recently published study among 45 men, showed that mpMRI and SelectMDx missed 3/9 (33.3%) and 4/9 (44.5%) clinically significant PCa, respectively.^11^ Furthermore, mpMRI combined with SelectMDx diagnosed 7/9 (77.8%) clinically significant PCa.^11^ Saturation prostate biopsies combined with MRI and ultrasound fusion biopsy outperformed significantly the diagnostic accuracy of mpMRI (84.5%) and SelectMDx (70.3%) in the diagnosis of clinically significant PCa.^11^

Our study is limited by the relatively small sample size and has included a selective cohort of patients who had have had a confirmed prostate cancer diagnosis with prostate biopsy as well as mpMRI and SelectMDx test and had additionally undergone a radical prostatectomy. Furthermore, biomarkers are mostly designed to predict the chance of clinically significant PCa (GS ≥7) found in transrectal or transperineal biopsy. This is also the case for SelectMDx that is designed and promoted to help patients decide if they need to have a prostate biopsy or in some cases a repeat prostate biopsy. However, the problem with ultra sound guided biopsy is that it not only has a false‐negative rate of approximately 20%,^12^, ^13^ but it also has difficulty detecting PCa in the anterior (and apical parts) of the prostate.^11^

In fact, what the patient and the treating doctor really want to know, is if the patient has a clinically significant PCa. The best answer to this question is given in the RP histology. Therefore, we believe that the ultimate answer comes for a study like ours that has compared the mpMRI and SelectMDx predictive values alone and compared that to the eventual RP histology which is the ultimate gold standard.

## 5 CONCLUSIONS

In conclusion, our study showed, that in this real‐life selected group of consecutive patients with a confirmed positive PCa biopsy and available mpMRI, the SelectMDx liquid biopsy test does not provide an advantage in the decision making of diagnostic work up and therapy of clinically significant PCa.

The addition of SelectMDx was only found valuable in those patients who had a very high‐risk PCa (ie, GS ≥8) with a negative mpMRI result.

## CONFLICT OF INTEREST

Dr Mohammad Sajjad Rahnama'i wants to declare speaker/consultant for Astellas, Dr Pfleger, Bioness and Janssen.

## AUTHORS' CONTRIBUTIONS

All authors had full access to the data in the study and take responsibility for the integrity of the data and the accuracy of the data analysis. *Conceptualization*, M.S.R., C.B., T.V.; *Methodology*, M.S.R., M.S.‐H., T.V.; *Investigation*, M.S.R., C.B., M.S.‐H., C.K., T.V.; *Formal Analysis*, M.S.R.; *Writing ‐ Original Draft*, M.S.R., C.B., M.S.‐H., C.K., T.V.; *Writing ‐ Review & Editing*, M.S.R., C.B., M.S.‐H., C.K., T.V.; *Supervision*, M.S.R., C.K., T.V.

## ETHICAL STATEMENT

This study was carried out in agreement with our local institutional ethical committee requirement and included patient consent.

## Data Availability

The raw data are available and can be required from the corresponding author.
